# Identification of sodium channel toxins from marine cone snails of the subgenera *Textilia* and *Afonsoconus*

**DOI:** 10.1007/s00018-023-04935-0

**Published:** 2023-09-09

**Authors:** Kirsten L. McMahon, Henrik O’Brien, Christina I. Schroeder, Jennifer R. Deuis, Dhananjeyan Venkatachalam, Di Huang, Brad R. Green, Pradip K. Bandyopadhyay, Qing Li, Mark Yandell, Helena Safavi-Hemami, Baldomero M. Olivera, Irina Vetter, Samuel D. Robinson

**Affiliations:** 1 Institute for Molecular Bioscience, The University of Queensland, St Lucia, QLD 4072 Australia; 2 Biology Department, University of Utah, Salt Lake City, UT 84112 USA; 3 Peptide Therapeutics, Genentech, 1 DNA Way, South San Francisco, CA 94080 USA; 4 Department of Human Genetics, Utah Center for Genetic Discovery, University of Utah, Salt Lake City, UT 84112 USA; 5 Cancer Bioinformatics, Huntsman Cancer Institute, University of Utah, Salt Lake City, UT 84112 USA

**Keywords:** Conotoxin, µ-conotoxin, Voltage-gated sodium channel, Historical DNA

## Abstract

Voltage-gated sodium (Na_V_) channels are transmembrane proteins that play a critical role in electrical signaling in the nervous system and other excitable tissues. µ-Conotoxins are peptide toxins from the venoms of marine cone snails (genus *Conus*) that block Na_V_ channels with nanomolar potency. Most species of the subgenera *Textilia* and *Afonsoconus* are difficult to acquire; therefore, their venoms have yet to be comprehensively interrogated for µ-conotoxins. The goal of this study was to find new µ-conotoxins from species of the subgenera *Textilia* and *Afonsoconus* and investigate their selectivity at human Na_V_ channels. Using RNA-seq of the venom gland of *Conus* (*Textilia*) *bullatus,* we identified 12 µ-conotoxin (or µ-conotoxin-like) sequences. Based on these sequences we designed primers which we used to identify additional µ-conotoxin sequences from DNA extracted from historical specimens of species from *Textilia* and *Afonsoconus*. We synthesized six of these µ-conotoxins and tested their activity on human Na_V_1.1–Na_V_1.8. Five of the six synthetic peptides were potent blockers of human Na_V_ channels. Of these, two peptides (BuIIIB and BuIIIE) were potent blockers of hNa_V_1.3. Three of the peptides (BuIIIB, BuIIIE and AdIIIA) had submicromolar activity at hNa_V_1.7. This study serves as an example of the identification of new peptide toxins from historical DNA and provides new insights into structure–activity relationships of µ-conotoxins with activity at hNa_V_1.3 and hNa_V_1.7.

**Supplementary Information:**

The online version contains supplementary material available at 10.1007/s00018-023-04935-0.

## Introduction

Voltage-gated sodium (Na_V_) channels are transmembrane proteins responsible for the initiation and propagation of action potentials in excitable cells. In humans, there are nine Na_V_ channel subtypes (Na_V_1.1–Na_V_1.9) with distinct expression profiles and functions [1]. Na_V_1.1, 1.2, 1.3 and 1.6 are predominately expressed in the central nervous system, Na_V_1.7, 1.8 and 1.9 in the peripheral nervous system, and Na_V_1.4 and Na_V_1.5 in skeletal muscle and cardiac tissue, respectively. Na_V_ channel dysfunction is associated with numerous neurological disorders, including epilepsy and chronic pain [2] and compounds that selectively modulate individual subtypes are important tools to study the function of Na_V_ channels and have the potential to serve as drug leads.

Numerous and diverse classes of toxins have evolved to target and modulate Na_V_ channels [3–5]. µ-Conotoxins are peptides from the venoms of marine snails of the genus *Conus* [6], which bind to “site 1” of Na_V_ channels, where they block Na^+^ conductance by occluding the channel pore [7]. They comprise 18–26 amino acid residues with six cysteine residues arranged in a CC–C–C–CC pattern and are derived from the “M gene superfamily” of conotoxins [8, 9].

The first µ-conotoxin described was GIIIA (along with several homologous peptides) from the venom of *Conus* (*Gastridium*) *geographus* Linnaeus, 1758 [6]. GIIIA blocked rat skeletal muscle Na^+^ current, with no discernible effect on that of nerve or brain, providing some of the earliest evidence of the existence of distinct Na_V_ subtypes and demonstrating the capacity of µ-conotoxins to selectively block these subtypes [6]. Since then, a number of other µ-conotoxins have been described from the venoms of other *Conus* species [10]. While the majority of these, like GIIIA, have a preference for the skeletal muscle Na_V_ channel (Na_V_1.4) over other subtypes, several “neuronal subtype-preferring” µ-conotoxins have also been described. For example, KIIIA from the venom of *Conus* (*Afonsoconus*) *kinoshitai* Kuroda, 1956 [11] was most potent at the neuronal subtype Na_V_1.2 over Na_V_1.4 and was also active at Na_V_1.7 (rat channels). Several efforts have been made to improve the selectivity and investigate the analgesic activity of KIIIA [12–15], and it has served as a useful tool in determining the three-dimensional structure of Na_V_1.2 [16]. BuIIIB from the venom of *Conus* (*Textilia*) *bullatus* Linnaeus, 1758 [17] was a potent blocker of rNa_V_1.3 [18]. As with KIIIA, efforts have been made to tune the selectivity of this peptide for Na_V_1.3 over Na_V_1.4 [19, 20].

µ-Conotoxins have so far been restricted to the venoms of fish-hunting and closely related lineages of *Conus*, and of these, the subgenera *Afonsoconus* and *Textilia* appear to be a promising source of “neuronal subtype-preferring” µ-conotoxins. However, for species from these subgenera other than *C.* (*T.*) *bullatus*, it is difficult to acquire live specimens for venom analysis. For example, *Conus* (*Textilia*) *dusaveli* (H. Adams, 1872) was, for more than a decade, known by a single shell found by a fisherman in the stomach of a fish caught at 60 fathoms (110 m) off Mauritius [21]. *Conus* (*Afonsoconus*) *bruuni* Powell, 1958 was formally described only in 1958, after its discovery from deep-water dredging by the Galathea expedition off the remote Pacific Kermadec islands [22].

In this study, we generated a venom gland transcriptome of *C.* (*T.*) *bullatus*, from which we identified several new µ-conotoxin sequences. We used these, and previously reported sequences, to design “µ-conotoxin-specific” primers which we used to amplify additional new µ-conotoxin sequences from DNA extracted from historical specimens of *C.* (*T.*) *dusaveli*, *Conus* (*Textilia*) *adamsonii* Broderip, 1836 and *C.* (*A.*) *bruuni*. We then synthesized several of the identified µ-conotoxins and profiled their activity at human Na_V_ channel subtypes Na_V_1.1 to Na_V_1.8.

## Methods

### Specimens

*C.* (*T.*) *bullatus* was collected from Caw-oy, Olango, the Philippines. A foot tissue sample of *C.* (*T.*) *adamsonsii* was acquired from the AAMP Malacology collection of the Muséum National D'histoire Naturelle, Paris. The sample was from a specimen (IM-2013-40001) collected at a depth of 6–12 m near Ua-Pou, Marquesas Islands, French Polynesia as part of the Pakaihi I Te Moana expedition, 2012. A foot tissue sample of *C.* (*A.*) *bruuni* was acquired from the Samadi, Lozouet and Castelin malacology collection of Muséum National d'Histoire Naturelle, Paris. The sample was from a specimen (IM-2009-18222) collected at a depth of 300–320 m in the Antigonia seamount, Norfolk Ridge, New Caledonia as part of the Terrasses expedition, 2008. A single specimen of *C.* (*T.*) *dusaveli* was sourced April 2006 from Balut Island, the Philippines. The whole body had initially been stored in 70% ethanol and was noticeably degraded before being transferred to 95% ethanol and stored at 4 °C.

For species names we have followed the classification proposed by Puillandre et al. [23].

### Venom gland transcriptome of *C. *(*T.*)* bullatus*

The whole venom gland of one adult specimen of *C.* (*T.*) *bullatus* was removed, immediately placed in RNALater and stored at −80 °C. Total RNA was isolated from the homogenized venom gland using TRIzol reagent following the manufacturer’s protocol (Life Technologies Corporation). Integrity of RNA was verified on a Bioanalyzer instrument (Agilent Technologies), and Illumina libraries were prepared by Cofactor Genomics. An indexed library was constructed with a mean insert size of 170 bp with the Illumina TruSeq mRNA Sample Prep Kit with oligo(dT) selection. 101 cycle paired-end sequencing was performed on an Illumina HiSeq2000 instrument (in a batch of six indexed samples). Adapter trimming of de-multiplexed raw reads was performed using fqtrim [24], followed by quality trimming and filtering using prinseq-lite [25]. Error correction was performed using the BBnorm ecc tool, part of the BBtools package. Trimmed and error-corrected reads were assembled using Trinity (version 2.2.1) [26] with k-mer length of 31 and minimum k-mer coverage of 10. Assembled transcripts were annotated using a blastx [27] search (*E* value setting of 1e−3) against a combined database derived from UniProt and Conoserver [28]. TPM counts were generated using the Trinity RSEM [29] plugin (align_and_estimate_abundance) and expression data analysed using the trinity utilities: abundance_estimates_to_matrix and contig_ExN50_statistic. Conotoxin-encoding transcripts were extracted, trimmed to open-reading frame and redundant, partial and/or poorly expressed transcripts (< 1 TPM) discarded. The fidelity of each assembled conotoxin transcript was then manually checked using the Map-to-Reference tool of Geneious, version 8.1.7 [30]. Erroneous transcripts were discarded, while unassembled variants were manually rebuilt and added back to the final data set. Relative TPM counts were generated using the Trinity RSEM plugin (align_and_estimate_abundance) and expression data analysed using the trinity utility abundance_estimates_to_matrix.

### DNA extraction

DNA was extracted from the foot tissue of *C.* (*T.*) *adamsonii, C.* (*A.*) *bruuni, C.* (*T.*) *bullatus* and* C.* (*T.*) *dusaveli* using the e.Z.N.A. Mollusc DNA Kit (OMEGA bio-tek), according to the manufacturer’s instructions. Quality and yield of extracted DNA was examined by gel electrophoresis and using an Epoch Microplate Spectrophotometer (BioTek). Extracted DNA was stored at −20 °C.

### PCR, cloning and sequencing

Primers for amplifying µ-conotoxins were designed based on conserved regions in the propeptide and 3′ UTR of *C.* (*T.*) *bullatus* µ-conotoxins, KIIIA (*C.* (*A.*) *kinoshitai*) and SIIIA (*C.* (*T.*) *striatus*). Primary PCR was performed using Advantage 2 DNA Polymerase (Clontech), sense primer 5′ TGCAGAGCGTATGCAGGA 3′ and antisense primer 5′ TCTGAGCAAGACTGCAATCAT 3′. Nested PCR was performed using Advantage 2 DNA Polymerase sense primer 5′ TGCAGGACGACATTTCATCT 3′ and antisense primer 5′ GACATTGATGTAGCCGTGATA 3′. Nested PCR products were diluted, ligated into pGEM-T Easy vector (Promega), and cloned in *E. coli* DH10B competent cells (Novagen). Bacteria containing the insert from nested PCR were grown overnight in LB with ampicillin. Plasmids were isolated using the Qiaprep Spin Miniprep Kit (Qiagen) and sequenced using T7 and SP6 primers at the University of Utah Sequencing Core Facility.

### Peptide synthesis

Synthetic BuIIIB, BuIIID, BuIIIE, AdIIIA, DIIIA and BIIIA were assembled by solid-phase peptide synthesis on a Liberty Prime automatic synthesizer (CEM), on rink amide-AM resin (0.1 mmol/g) using 9-fluorenylmethoxycarbonyl (Fmoc) methodology. Peptides were cleaved from solid support and side chains simultaneously deprotected in 92.5% TFA (trifluoracetic acid)/2.5% triisopropylsilane/2.5% H_2_O/2.5% 3,6-dioxa-1,8-octanedithiol for 2 h at room temperature. Excess TFA was evaporated by N_2_ flow, followed by peptide precipitation in ice-cold diethyl ether and centrifugation. Peptides were redissolved in 50% acetonitrile and lyophilized prior to oxidation.

DIIIA was thermodynamically oxidized in 0.1 M ammonium biocarbonate pH 7.5, 100× GSH and 10× GSSG for 2 h with stirring. BuIIIB, BuIIID, BuIIIE, AdIIIA, and BIIIA were synthesized with protecting groups trityl (Trt), acetamidomethyl (Acm) and 4,4′-dimethylsulfinylbenzhydryl (Msbh) with connectivity CysI–CysIV (Msbh)/CysII–CysV (Acm)/CysIII–CysVI (Trt) for regioselective oxidation. The peptides (2 mg/mL) were dissolved in acetic acid and stirred at room temperature. Five equivalents of free Trp were added to minimize side-reactions with iodine. I_2_ solution (20 mg/mL) was added dropwise until the reaction turned pale-yellow and the color persisted. Following 15 min of stirring, the reaction was diluted with H_2_O and hydrochloric acid to a final concentration of 50% acetic acid (v/v) and 1% hydrochloric acid (v/v). Next, eight equivalents of I_2_ were added to the reaction and stirred for a further 30 min. The reaction was quenched with aqueous ascorbic acid (10 mM) until the solution became colorless. The intermediate product was isolated by reversed phase-high-performance liquid chromatography (RP-HPLC) and fractions containing the desired product were identified by electrospray ionization–mass spectrometry (ESI–MS), pooled and lyophilized. To remove the Msbh protecting groups and form the final disulfide bond, peptides were dissolved in TFA (1 mg/mL) and cooled on ice. Five equivalents of free Trp, dimethyl sulfate (1% v/v) and 20 equivalents of NaI were added and stirred for 15 min. The reaction was quenched by diluting with aqueous ascorbic acid (10 nM; 15 × the initial volume of TFA). The final product was isolated by RP-HPLC using a Phenomenex, 5 µm C18 110 Å, 250 × 5 mm column and a linear gradient 5–25% over 40 min. Fractions containing the desired product confirmed by ESI–MS were pooled, lyophilized and stored at −20 °C. Analytical RP-HPLC and ESI–MS were used to confirm the purity and mass, respectively, of each of the folded products.

BIIIA was also synthesized as above with the alternative cysteine framework CysI–CysV (Msbh), CysII–CysIV (Acm), CysIII–CysVI (Trt). This synthetic isomer is referred to as BIIIA-iso2.

### Whole-cell patch-clamp electrophysiology

Activity was assessed in Human Embryonic Kidney (HEK) 293 cells stably expressing the α-subunit of human Na_V_1.1–Na_V_1.7 plus the β1 subunit (SB Drug Discovery) and Chinese Hamster Ovary (CHO) cells stably expressing human Na_V_1.8 plus the β3 subunit in a tetracycline-inducible system (ChanTest). Cells were maintained on Mimium Essential Medium Eagle supplemented with 10% Foetal Bovine Serum, 2 mM l-glutamine, and selection antibiotics as previously described [31], in an incubator at 37 °C with 5% CO_2_ and passaged every 3–4 days (at 70–80% confluency) using TrypLE Express (Thermo Fisher Scientific).

Whole-cell patch-clamp experiments were performed using a QPatch16 II automated electrophysiology platform (Sophion Bioscience, Ballerup, Denmark) using single-hole (QPlate 16 with a standard resistance of 2 ± 0.4 MΩ) or multi-hole (QPlate 16X with a standard resistance 0.2 ± 0.04 MΩ, Na_V_1.8 only). Whole-cell currents were filtered at 8 kHz and acquired at 25 kHz and the linear leak was corrected by P/4 subtraction.

The extracellular solution (ECS) contained 145 mM NaCl (replaced with 70 mM choline chloride, 70 mM NaCl for Na_V_1.4, Na_V_1.5 and Na_V_1.7), 4 mM KCl, 2 mM CaCl_2_, 1 mM MgCl_2_, 10 mM HEPES, and 10 mM glucose (pH 7.4; osmolarity 305 mOsm). The intracellular solution (ICS) contained 140 mM CsF, 1/5 mM EGTA/CsOH, 10 mM HEPES, and 10 mM NaCl (pH 7.3; osmolarity 320 mOsm). TTX (1 μM) was added to the ECS for Na_V_1.8 recordings to inhibit background endogenous TTX-sensitive current in CHO cells. Peptides were diluted in ECS with 0.1% Bovine Serum Albumin (BSA). Concentration–response experiments were performed using a holding potential of −90 mV and a 50-ms pulse to −20 mV (+ 10 mV for Na_V_1.8) every 20 s (0.05 Hz), after a 5-min incubation of each concentration of peptide. Concentration–response curves were generated by normalizing peak current to buffer control and fitted to a four-parameter Hill equation with variable Hill coefficient: *y* = Bottom + (Top–Bottom)/(1 + 10^(LogIC50 − *x*) × HillSlope)^).

### Statistics

Data were plotted and analysed using Prism v9.0.0 (GraphPad Software). Statistical significance was defined as *P* < 0.05. All data are presented as mean ± SEM. Selectivity profiles are based on differences in potency determined by ordinary one-way ANOVA with Tukey’s multiple comparisons test.

### Data availability

The nucleotide sequences of Bu3.4 (BuIIID), Bu3.8 (BuIIIE), Bu3.5, Bu3.6, Bu3.7, Bu3.9, Bu3.10, Bu3.11, Bu3.12, D3.1, Ad3.1 (including its intronic sequence) and B3.1 have been deposited at DDBJ/EMBL/GenBank [Accessions: OR128446–OR128457]. Sequences of all additional conotoxins identified in the venom gland transcriptome of *C. (T.) bullatus* are provided as Supplementary file 2.

## Results

### Diverse μ-conotoxin-encoding transcripts are expressed in the venom gland of *C. (T.) bullatus*

**Fig. 1:**
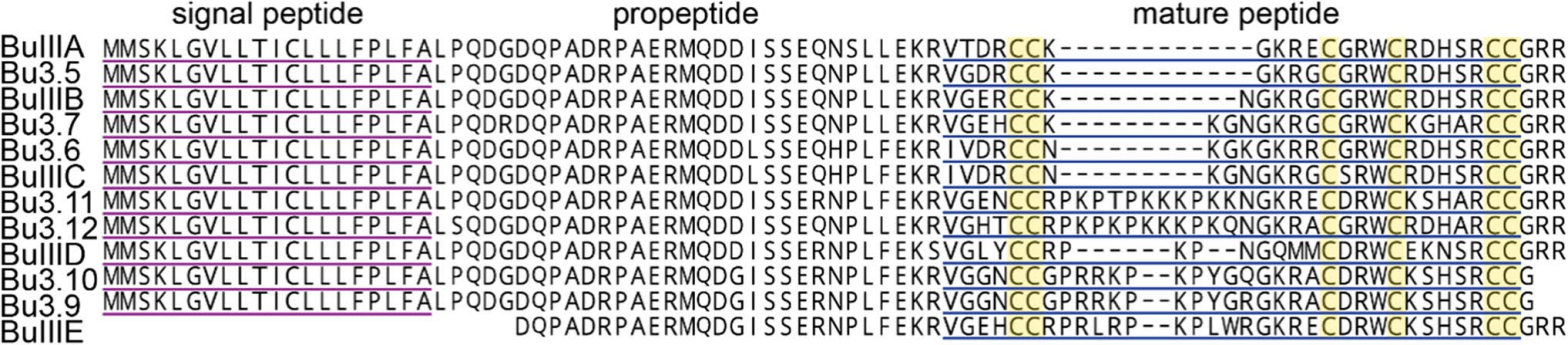
Diverse μ-conotoxin-encoding transcripts are expressed in the venom gland of *C.* (*T.*) *bullatus*. Signal peptides and predicted mature peptides are underlined in purple and blue, respectively. Cysteine residues of the mature peptides are highlighted yellow

Using RNA-seq we generated a transcriptome of the venom gland of *C.* (*T.*) *bullatus* and used it to search for transcripts encoding M-superfamily conotoxins. We identified a total of 12 putative M-superfamily μ-conotoxin-encoding sequences (Fig. 1), including the previously described BuIIIA, BuIIIB, BuIIIC [17] and an orthologue of Bu17 (23/26 sequence identity) [32]. Other conotoxins identified in the venom gland transcriptome are listed in Supplementary file 2. Each of the 12 putative M-superfamily μ-conotoxin-encoding sequences encoded putative mature peptides that shared the typical sequence features of other μ-conotoxins, i.e., the CC–C–C–CC pattern (framework III), a four residue *N*-terminal ‘tail’ (predicted on the basis of the dibasic propeptide cleavage sites (KR) of each precursor), amidated *C*-termini (predicted on the basis of the *C*-terminal amidating enzyme recognition motif motif (G or GRR) of each precursor) and three and five residues in their intercysteine loops 2 and 3, respectively. A conspicuous feature was the diversity in the length of intercysteine loop 1, which ranged from four residues in BuIIIA to 17 residues in Bu3.11 and Bu3.12.

### Extraction of DNA and sequencing of μ-conotoxin sequences from museum specimens

**Fig. 2:**
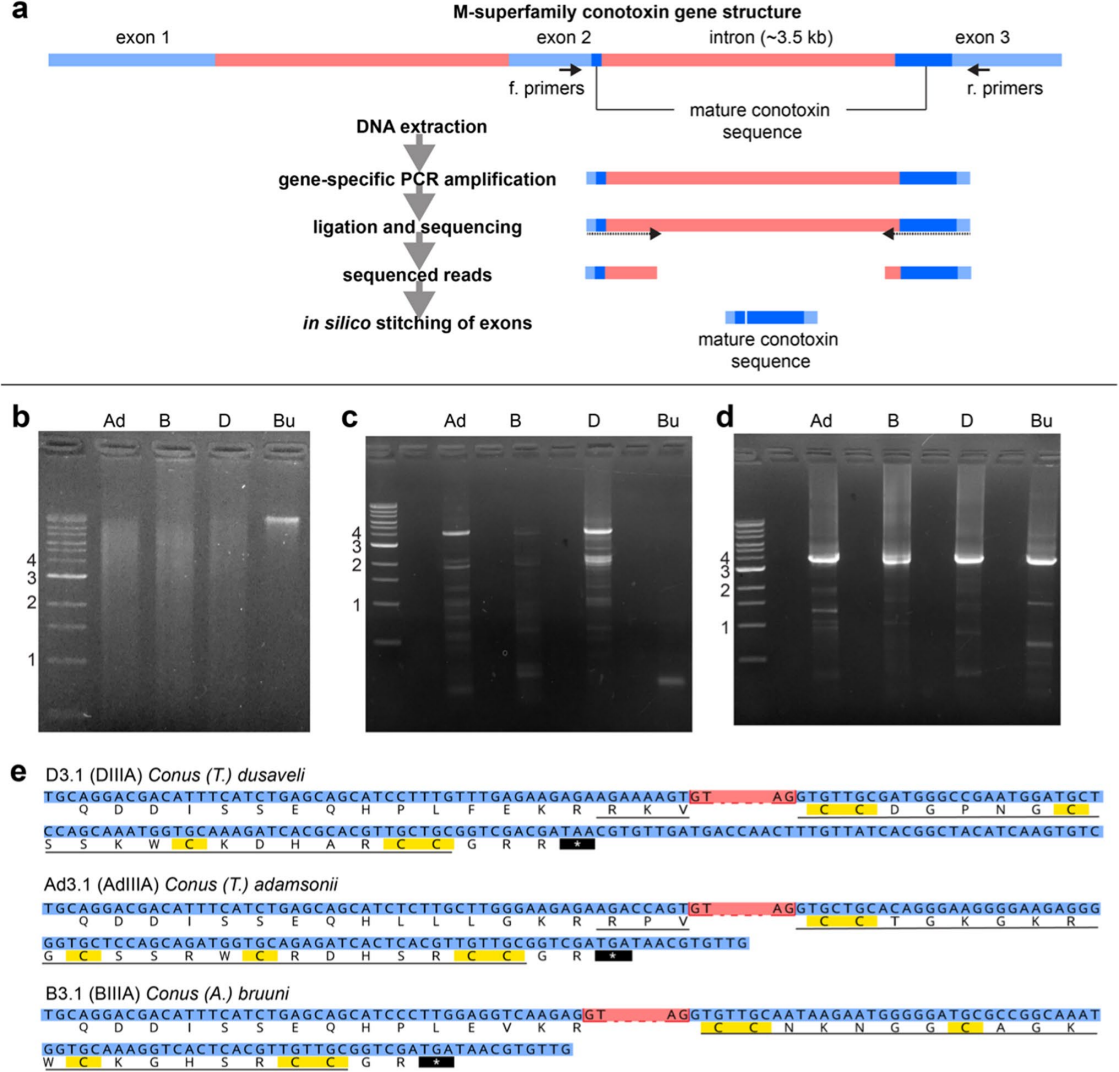
DNA extraction, targeted amplification and sequencing of μ-conotoxins. **a** Experimental design: the complete precursor sequences of M-superfamily conotoxins span 3 exons, divided by two introns [33]. The mature conotoxin sequence is split between exons 2 and 3. Primers were designed targeting exon 2 and exon 3 to amplify sequences encoding μ-conotoxin mature peptides from extracted DNA. PCR products were ligated into a vector, transformed and sequenced. Complementary sequenced reads were stitched in silico to give the complete putative μ-conotoxin mature peptide sequence. **b** Gel electrophoresis of DNA extracted from *Conus* tissue samples. From left to right: *C.* (*T.*) *adamsonii, C.* (*A.*) *bruuni, C.* (*T.*) *dusaveli, C.* (*T.*) *bullatus* (100 μg of DNA loaded for each). All DNA, except from *C.* (*T.*) *bullatus*, was extracted from foot tissue that had been stored in 95% ethanol at room temperature for 5–11 y. *C.* (*T.*) *bullatus* DNA was extracted from a venom gland stored in RNAlater at −80 °C for ~ 1 y. **c** Gel electrophoresis of first round PCR products. From left to right: *C.* (*T.*) *adamsonii, C.* (*A.*) *bruuni, C.* (*T.*) *dusaveli, C.* (*T.*) *bullatus*. **d** Gel electrophoresis of nested PCR products, each showing a strong band at ~ 3.5 kb. From left to right: *C.* (*T.*) *adamsonii, C.* (*A.*) *bruuni, C.* (*T.*) *dusaveli, C.* (*T.*) *bullatus*. **e** μ-conotoxin sequences detected from *C.* (*T.*) *adamsonii, C.* (*A.*) *bruuni* and *C.* (*T.*) *dusaveli*. Exon nucleotide sequence are colored blue, while intervening intron nucleotide sequence are colored red. Intron sequence has been removed for clarity showing only the first two nucleotides at each boundary. Translated sequences are shown below each nucleotide sequence, with the predicted mature peptides underlined. Cysteine residues and stop codons, in the translated sequences are highlighted yellow and black, respectively

We used a sequence alignment of previously reported μ-conotoxin transcripts, including those of *C.* (*T.*) *bullatus*, to design multiple sets of μ-conotoxin gene-specific primers (see “Methods” section). Forward and reverse primers were designed to complement the propeptide and 3′-UTR regions, respectively, bounding the predicted μ-conotoxin mature peptide region (Fig. 2a).

We extracted DNA from ~ 30 mg of foot tissue from historical specimens of *C.* (*A.*) *bruuni* and *C.* (*T.*) *adamsonii* (provided by the Muséum National d'Histoire Naturelle, Paris, collected in 2008 and 2012, respectively, and stored at room temperature in 95% ethanol) and *C.* (*T.*) *dusaveli* (collected in 2006 and stored at room temperature in 70% ethanol). *C.* (*T.*) *bullatus* venom gland tissue (stored in RNAlater at −80 °C for ~ 1 y) was used as a positive control. DNA extracted from each ethanol-stored foot tissue produced a smear across the complete molecular weight range when examined by gel electrophoresis, while that from the venom gland of *C.* (*T.*) *bullatus* produced a single sharp high molecular weight band (Fig. 2b).

The first round of PCR (30 cycles) for each extracted DNA sample, yielded multiple products ranging from 1 to 4 kb (Fig. 2c). We performed a second round of “nested” PCR (30 cycles), which yielded major products of ~3.5 kb for each sample.

Nested PCR products (~ 3.5 kb) were ligated into pGEM-T Easy vector for Sanger sequencing. Sequencing of the ligated vector was performed using T7 and SP6 primers to sequence from each end of each ~ 3.5 kb PCR product. Using this approach, we were able to sequence for each PCR product, one stretch that contained part of exon 2 (which included part of the propeptide, and for two of the three conotoxins, the *N*-terminus of the mature peptide) and a complementary stretch which contained part of exon 3, including the remainder of each mature peptide as well as some of the 3′ UTR. With additional iterative primer design sequencing we sequenced the complete intervening intron of the sequence from *C.* (*T.*) *adamsonii* (Ad3.1) confirming a length of 3477 bases (Supplementary Fig. S1), consistent with the size of the nested PCR product (~ 3.5 kb). We analysed each sequence for exon/intron and intron/exon boundaries and “stitched” the exons together to yield the short stretch of contiguous open-reading frame encoding each μ-conotoxin mature peptide. The sequences were translated and *N*- and *C*-terminal peptide maturation sites predicted on the basis of alignments with previously described μ-conotoxin sequences, yielding the putative μ-conotoxin mature peptide sequences. For each species (*C.* (*A.*) *bruuni*, *C.* (*T.*) *adamsonii* and *C.* (*T.*) *dusaveli*), a single putative μ-conotoxin sequence was amplified: D3.1 (DIIIA) from *C.* (*T.*) *dusaveli*, Ad3.1 (AdIIIA) from *C.* (*T.*) *adamsonii* and B3.1 (BIIIA) from *C.* (*A.*) *bruuni* (Fig. 2e).

**Fig. 3:**
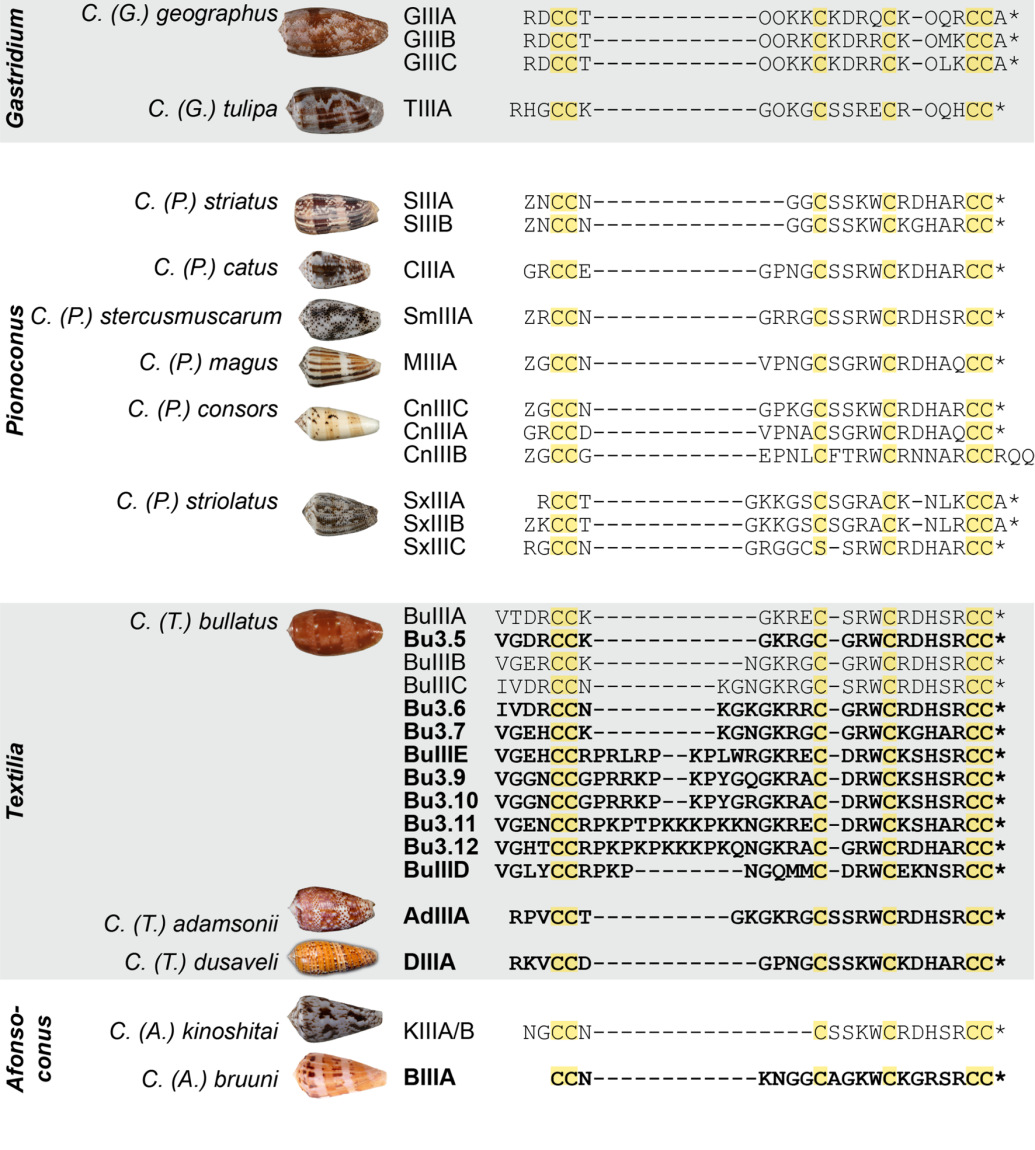
Alignment of μ-conotoxin mature peptide sequences. Sequences reported in this study are in bold. Cysteine residues are highlighted yellow. Z, *N*-terminal pyroglutamate; O, hydroxyproline; *, *C*-terminal amidation

The mature peptide sequence of each of the three new putative μ-conotoxins had a type III cysteine framework (CC–C–C–CC). DIIIA and AdIIIA each had an *N*-terminal extension of three residues, while BIIIA lacked the *N*-terminal extension (i.e., two to four residues preceding the first cysteine) seen in all other μ-conotoxins. On the basis of the presence of an amidating enzyme recognition motif (GR or GRRR) at the *C*-terminus of all three sequences, each were predicted to exist in the respective venoms with a *C*-terminal amidation. A comparison of the mature peptide sequences of each of the new putative μ-conotoxins from *C.* (*T.*) *bullatus*, *C.* (*A.*) *bruuni*, *C.* (*T.*) *adamsonii* and *C.* (*T.*) *dusaveli* to previously reported μ-conotoxins is shown in Fig. 3. DIIIA is very similar to CIIIA from the venom of *Conus* (*Pionoconus*) *catus* Hwass in Bruguière, 1792, sharing 18 of 22 aligned residues, with the only major difference occurring at the *N*-terminus. AdIIIA shares 100% identity in intercystine loops 2 and 3 (nine residues) with SmIIIA form *Conus* (*Pionoconus*) *stercusmuscarum* Linnaeus, 1758, while its loop 1 sequence resembles that of BuIIIC from *C.* (*T.*) *bullatus*. BIIIA shows no obvious similarity to other μ-conotoxins, including KIIIA/B from the venom of consubgeneric *C.* (*A.*) *kinoshitai*.

### Synthesis and regioselective folding of μ-conotoxins

We selected six of the peptides (BuIIIB, BuIIID, BuIIIE, AdIIIA, DIIIA and BIIIA) for testing at Na_V_ channels and prepared these using solid phase peptide synthesis. We initially attempted undirected thermodynamic folding of AdIIIA, DIIIA and BIIIA; however, using this strategy, of the three peptides only DIIIA folded into a single well-defined major product.

With three disulfides, μ-conotoxins can theoretically fold into 15 possible cystine connectivities. Where examined, native μ-conotoxins have a cystine connectivity of CysI–CysIV, CysII–CysV, CysIII–CysVI [34, 35], as do active synthetic μ-conotoxins [36, 37] (although synthetic KIIIA, under certain in vitro folding conditions favours, and is also active, with a CysI–CysV, CysII–CysIV, CysIII–CysVI connectivity) [38]. Thus, we prepared AdIIIA, BIIIA, BuIIIB, BuIIID and BuIIIE using a regioselective approach, whereby each Cys in the disulfide pair, CysI–CysIV, CysII–CysV, CysIII–CysVI, were protected with Msbh, Acm or Trt, respectively. This enabled formation of disulfides in a stepwise fashion resulting in folded peptides with the canonical disulfide connectivity.

### µ-Conotoxins BuIIIB, BuIIIE, DIIIA, AdIIIA and BIIIA have distinct selectivity profiles for human Na_V_ channel subtypes

We used automated whole-cell patch-clamp electrophysiology to determine the potency of each of the peptides at human Na_V_1.1–1.8.

The activity of BuIIIB has been investigated at rodent Na_V_ channels [18], but selectivity at human Na_V_ channels has not previously been reported. Here, BuIIIB was most potent at hNa_V_1.3 (IC_50_ = 5 ± 1 nM, *n* = 4 cells) and hNa_V_1.4 (IC_50_ = 11 ± 0.5 nM, *n* = 4 cells), with ~ 5–10-fold selectivity over hNa_V_1.7 (IC_50_ = 55 ± 15 nM, *n* = 9 cells), hNa_V_1.1 (IC_50_ = 59 ± 24 nM, *n* = 3 cells) and hNa_V_1.2 (IC_50_ = 79 ± 13 nM, *n* = 5 cells) and > 30-fold selectivity over hNa_V_1.6 (IC_50_ = 171 ± 53 nM, *n* = 4 cells) (selectivity profile: 1.3 ≈ 1.4 < 1.7 ≈ 1.1 ≈ 1.2 ≈ 1.6). BuIIIB was inactive at hNa_V_1.5 and hNa_V_1.8 when tested up to 10 µM.

BuIIIE shared a similar selectivity profile (1.3 ≈ 1.4 < 1.1 ≈ 1.2 ≈ 1.6 ≈ 1.7 <  < 1.5) to BuIIIB but was less potent at all subtypes tested. BuIIID, up to a concentration of 10 μM, had no effect on hNa_V_1.4 or hNa_V_1.7 current (Table 1), and was, therefore, not tested on other subtypes. A difference between BuIIID and all described μ-conotoxins, is the negatively charged residue (Glu) at position 21. In all μ-conotoxins described to date, this residue is a positively charged Arg or Lys. This charged residue has been identified as a key contributor to µ-conotoxin Na_V_ channel inhibition [13], and may explain the observed lack of inhibitory activity for BuIIID.

**Table 1:** Potency (nM) of µ-conotoxins at human Na_V_ channel subtypes. Data are mean ± SEM

nM	Na_V_1.1	Na_V_1.2	Na_V_1.3	Na_V_1.4	Na_V_1.5	Na_V_1.6	Na_V_1.7	Na_V_1.8
BuIIIB	59 ± 24	79 ± 13	5 ± 1	11 ± 0.5	> 10,000	171 ± 53	55 ± 15	> 10,000
BuIIID	n.d	n.d	n.d	> 10,000	n.d	n.d	> 10,000	n.d
BuIIIE	911 ± 213	643 ± 167	121 ± 36	193 ± 72	7555 ± 2014	503 ± 83	633 ± 185	> 10,000
DIIIA	749 ± 76	1971 ± 238	185 ± 27	216 ± 45	> 10,000	1117 ± 451	7900 ± 1969	> 10,000
AdIIIA	96 ± 8	587 ± 97	163 ± 30	26 ± 2	> 10,000	1479 ± 223	137 ± 15	> 10,000
BIIIA	557 ± 116	167 ± 27	5172 ± 1828	595 ± 45	> 10,000	> 10,000	3304 ± 823	> 10,000
BIIIA-iso2	568 ± 179	398 ± 17	8316 ± 1205	78 ± 12	> 10,000	> 10,000	2486 ± 572	> 10,000

n.d., not determined

**Fig. 4:**
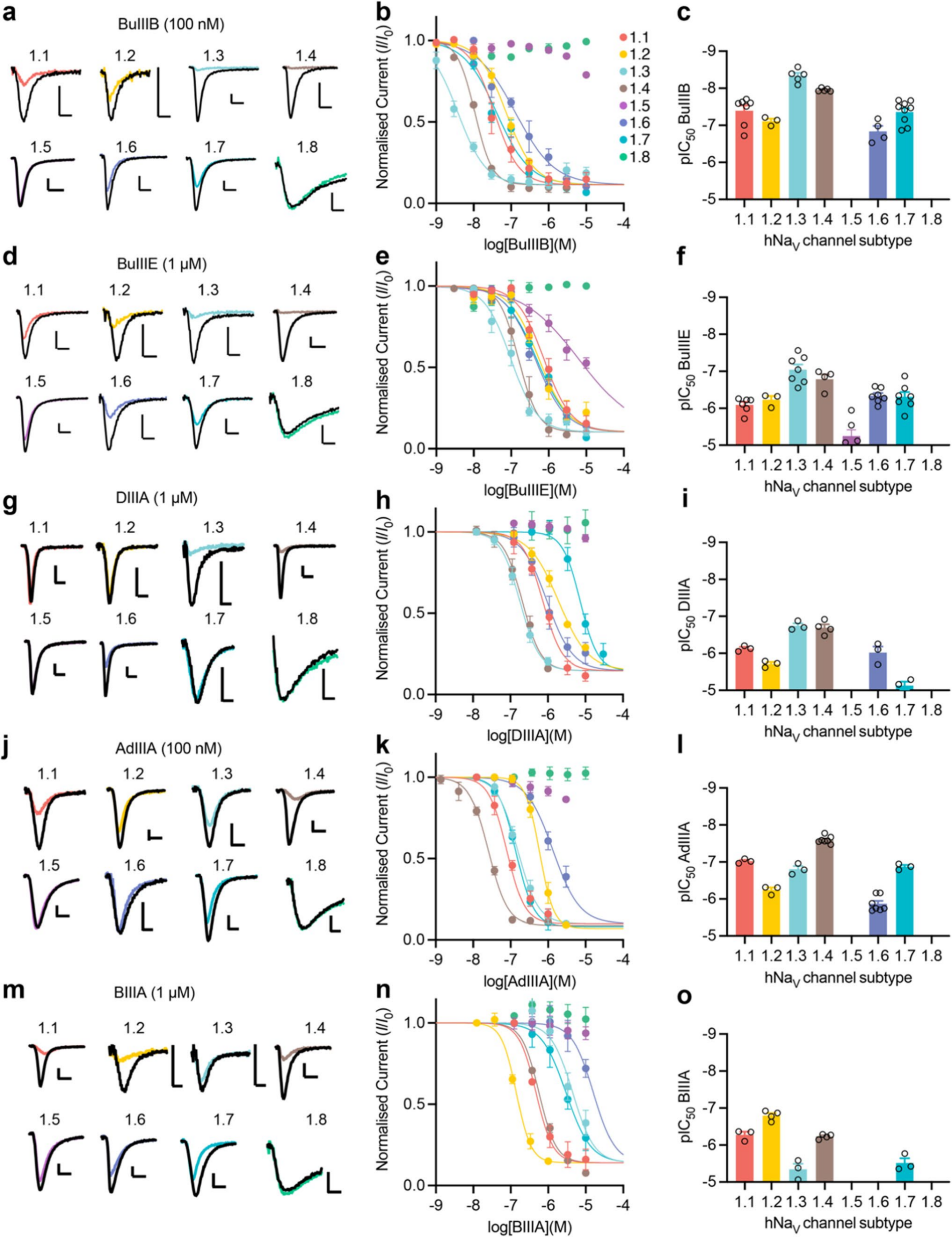
µ-Conotoxins BuIIIB, BuIIIE, DIIIA, AdIIIA and BIIIA have distinct selectivity profiles for human Na_V_ channel subtypes. **a** Representative traces for human Na_V_ channel subtypes 1.1–1.8 in the presence of BuIIIB (100 nM). **b** Concentration–response curves for BuIIIB at human Na_V_ channel subtypes 1.1–1.8. **c** pIC_50_ for BuIIIB at human Na_V_ channel subtypes 1.1–1.8. **d**–**f** equivalent data for BuIIIE. **g**–**i** Equivalent data for DIIIA. **j**–**l** Equivalent data for AdIIIA. (m–o) Equivalent data for BIIIA. Data are mean ± SEM

DIIIA was most potent at hNa_V_1.3 (IC_50_ = 185 ± 3 nM, *n* = 3 cells) and hNa_V_1.4 (IC_50_ = 216 ± 45 nM, *n* = 4 cells), and was also active at hNa_V_1.1, hNa_V_1.6 and hNa_V_1.2 (1.3 ≈ 1.4 < 1.1 ≈ 1.6 ≈ 1.2) but inactive (up to 10 µM) at hNa_V_1.5, hNa_V_1.7 and hNa_V_1.8 (Fig. 4, Table 1). AdIIIA was most potent at hNa_V_1.4 (IC_50_ = 26 ± 2 nM, *n* = 7 cells) with a selectivity profile of (1.4 < 1.1 ≈ 1.7 ≈ 1.3 < 1.2 < 1.6) and was inactive (up to 10 µM) at hNa_V_1.5 and hNa_V_1.8 (Fig. 4, Table 1). BIIIA was most potent at hNa_V_1.2 (IC_50_ = 167 ± 27 nM, *n* = 4 cells) with ~ 3.5-fold selectivity over hNa_V_1.1 and hNa_V_1.4 and a selectivity profile of (1.2 < 1.1 ≈ 1.4 < 1.7 ≈ 1.3). It was inactive (up to 10 µM) at hNa_V_1.5, hNa_V_1.6 and hNa_V_1.8 (Fig. 4, Table 1).

**Fig. 5:**
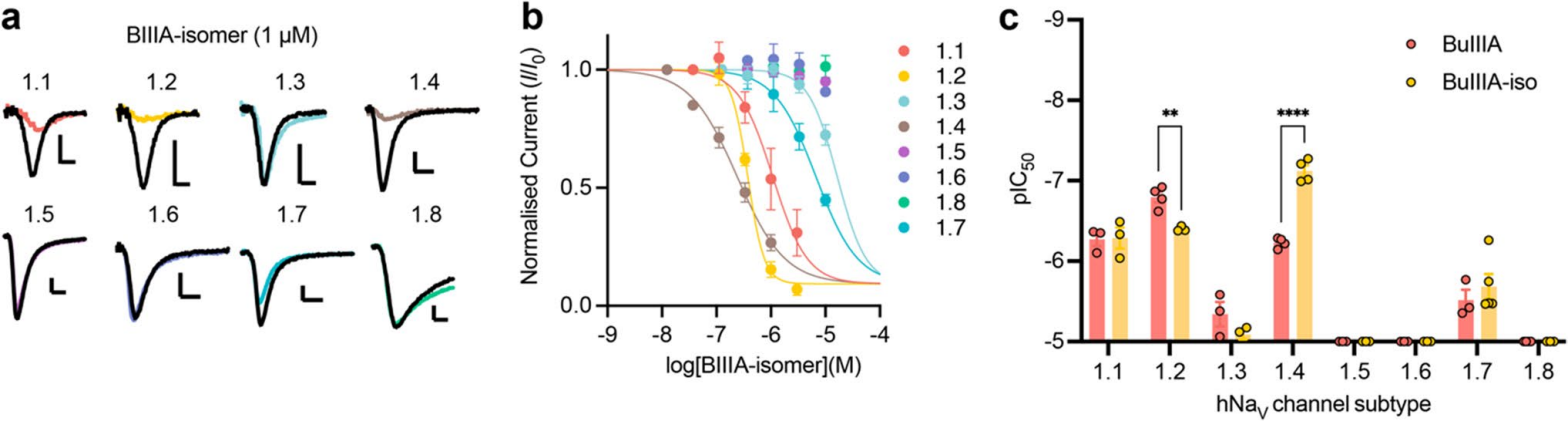
[CysI–CysV, CysII–CysIV, CysIII–CysVI] isomer (BIIIA-iso2) of µ-conotoxin BIIIA has subtle changes in its selectivity profile for human Na_V_ channel subtypes. **a** Representative traces for human Na_V_ channel subtypes 1.1–1.8 in the presence of BIIIA-iso2 (1 µM). **b** Concentration–response curves for BIIIA-isomer at human Na_V_ channel subtypes 1.1–1.8. **c** Comparison of pIC_50_ for BIIIA and BIIIA-iso2 at human Na_V_ channel subtypes 1.1–1.8. Data are mean SEM. **P < 0.01; ****P < 0.0001; unpaired *t* test

The BIIIA[CysI–CysV, CysII–CysIV, CysIII–CysVI] isomer (BIIIA-iso2) had a similar activity profile to BIIIA, but with increased potency at hNa_V_1.4 (IC_50_ = 78 ± 12 nM, *n* = 4 cells) and decreased potency at hNa_V_1.2 (IC_50_ = 398 ± 17 nM, *n* = 3 cells) (Fig. 5, Table 1) and the selectivity profile 1.4 < 1.2 ≈ 1.1 < 1.7 < 1.3.

## Discussion

Venoms are a promising source of novel peptides with potential utility as research tools and/or therapeutics [39]. RNA-seq of venom-producing tissues has become a common strategy for the identification of new venom peptides [40]. In this study, using RNA-seq we identified a total of 12 μ-conotoxin/μ-conotoxin-like sequences from *C.* (*T.*) *bullatus*. This included three of the four previously reported sequences (BuIIIA, BuIIIB and BuIIIC) [17] and a closely related homologue of Bu17 [32] (BuIIID). The additional sequences reported in this study, compared with the previous two studies, may reflect the greater depth of sequencing of our RNA-seq approach, although we cannot rule out other possibilities, such as intraspecific variation. Similarly, differences in sequencing technology may explain why 12 μ-conotoxin/μ-conotoxin-like sequences were detected in *C.* (*T.*) *bullatus*, while only a single sequence was detected in each of the other species investigated using our Sanger sequencing approach. However, we cannot rule out other factors, including the possibility that these species have only one μ-conotoxin/μ-conotoxin-like sequence, or that our approach was biased for the detection of individual sequences due to, e.g., differences in amplification rates. Future studies using different sequencing technology will be necessary to resolve these questions.

While high-output RNA-seq clearly has advantages over lower output methods for the identification of novel sequences, it still suffers from several limitations including a greater error rate compared with Sanger sequencing (which can lead to overestimation of sequence diversity [41]) and the poor performance of k-mer-based assembly algorithms in assembling multi-copy transcript families from short reads [42]. Indeed, with default settings, Trinity assembled only three of the 12 sequences correctly (BuIIIA, BuIIIC and Bu3.11). Only after remapping these sequences using the map-to-reference-tool of Geneious [30], did we identify and validate 12 sequences.

The most obvious limitation of an RNA-seq approach is the requirement for intact RNA–RNA rapidly degrades in non-living tissue, and RNA-seq approaches are, therefore, not possible in the absence of fresh tissue. While we could not obtain fresh or adequately preserved venom gland tissues for species of the subgenera *Afonsoconus* or *Textilia* other than *C.* (*T.*) *bullatus*, we were able to obtain tissue samples from historical specimens. DNA is more stable than RNA and from these specimens we were able to extract intact DNA and from this, identify μ-conotoxins. The study of DNA from historical museum specimens has found a number of applications in biology [43]. In this study we were “bioprospecting” for molecules with potential utility in physiology and medicine. As shown here, this approach is useful for identifying specific sequences of interest from samples that are otherwise unavailable, e.g., rare, endangered or extinct organisms. However, it is subject to some limitations: the specimen must be available in a collection and while we did not explicitly test this here, there are limitations associated with the age and storage conditions of specimens. Here, we were targeting a relatively short stretch of sequence ~ 1500 b, from samples that had been stored in ethanol (various concentrations) for a maximum of 11 y. Increased DNA fragmentation would be expected for older or poorly stored samples.

This approach is not suitable for the de novo identification of protein sequences. Prior knowledge of homologous sequences was critical in enabling the design of primers and prediction of mature peptide sequences and post-translational modifications (PTMs) (e.g., *C*-terminal amidation) including prediction of cystine connectivity. In this study sufficient information from homologous sequences facilitated our prediction of the *N*- and *C*-termini of the mature peptides. Similarly, on the basis of previous studies it was facile to predict amidation of the peptide *C*-termini. Several μ-conotoxins have one or more proline to hydroxyproline post-translation modifications [6, 36, 44]; each of the μ-conotoxins in this study, except BIIIA, have one or more proline residues in their sequence and it is possible that the native form of the toxins in the respective venoms also have these modifications; however, we were unable to make any confident predictions in this respect, and each conotoxin was synthesized without this PTM. Another important PTM is disulfide bond formation. Of the native μ-conotoxins for which disulfide connectivity has been investigated all have a cystine connectivity of CysI–CysIV, CysII–CysV, CysIII–CysVI [34, 35], as do active synthetic μ-conotoxins [36, 37]. On the basis of these studies, we synthesized our μ-conotoxins with this “canonical” cystine connectivity. However, it remains possible that the native forms in the venom may have a different cystine connectivity. For example, KIIIA, which under certain in vitro folding conditions favours a CysI–CysV, CysII–CysIV, CysIII–CysVI connectivity is more potent at rNa_V_1.2 and hNa_V_1.2 than the peptide with the canonical cystine connectivity [38, 45]. In this study, we synthesized two isomers of BIIIA, one with the canonical cystine connectivity and one with the alternative CysI–CysV, CysII–CysIV, CysIII–CysVI connectivity. Both isomers were active, the major difference being reduced potency at hNa_V_1.2 and increased potency at hNa_V_1.4 for the isomer with the alternative connectivity. These data provide more evidence that at least some μ-conotoxins can tolerate the aforementioned change in cystine connectivity with relatively subtle, but potentially important changes to their selectivity profiles.

The selectivity profile of BuIIIB at human Na_V_ channels is distinct from that reported in previous studies. Previous studies using rodent Na_V_ channels reported a selectivity profile of 1.4 < 1.2 < 1.3 < 1.1 < 1.6 < 1.5 with potencies (*K*_d_) of 3.6 and 200 nM for rNa_V_1.4 and rNa_V_1.3, respectively [18]. Here, in human Na_V_ overexpression systems, we observed a selectivity profile for BuIIIB of 1.3 ≈ 1.4 < 1.7 ≈ 1.1 ≈ 1.2 ≈ 1.6, with potencies of 5 and 11 nM for rNa_V_1.4 and rNa_V_1.3, respectively. Some of the earliest studies of μ-conotoxins reported differences between channels of different species. For example, KIIIA blocked TTX-resistant Na^+^ currents in frog (*Rana pipiens*) neuronal preparations, but had limited effects on action potentials in skeletal muscle (TTX-sensitive) [11]. In a subsequent study, using rat channels, KIIIA did not inhibit TTX-resistant subtypes, but potently inhibited TTX-sensitive subtypes with a selectivity profile of 1.2 < 1.4 < 1.7 ≈ 1.1 < 1.3 [46]. Similar differences have been observed with other μ-conotoxins [47]. Furthermore, KIIIA has a distinct selectivity profile for human Na_V_ channels (1.4 < 1.1 < 1.2 < 1.7 < 1.6 < 1.3) [48]. It is possible that these differences arise from the use of different techniques (e.g., two-electrode voltage- or patch-clamp methods on oocytes vs. mammalian cells) or different Na_V_ channel auxiliary subunit composition [49]; however, the most congruent explanation is that there are differences in the structure of the μ-conotoxin binding site between homologous channels of different species.

While there are differences in subtype selectivity profiles, all of the μ-conotoxins described so far, including those presented in this study, invariably share potent activity at Na_V_1.4. When viewed from a drug discovery perspective, activity at Na_V_1.4 is typically considered undesirable and most efforts to engineer μ-conotoxins have focused on increasing selectivity for potential therapeutic targets, e.g., Na_V_1.7 or Na_V_1.3 over Na_V_1.4 [10]. However, it is important to consider that in some systems, e.g., the peripheral and central nervous systems, Na_V_1.4 is absent. When this is taken into consideration, certain μ-conotoxins may find utility as research tools in delineating the physiological roles of specific Na_V_ channel subtypes in certain isolated systems. For example, BuIIIB, with an IC_50_ of 5 ± 1 nM at hNa_V_1.3, is ~ tenfold selective over other neuronal hNa_V_ subtypes. BuIIIB has the potential to serve as a tool for investigating the role of Na_V_1.3 in the human nervous system, and as a template for engineering more selective hNa_V_1.3 blockers.

Together, this study contributes new information on μ-conotoxin structure–activity relationship (SAR): BuIIID informs on the sequence features that drive activity at Na_V_ channels in general; BuIIIB, BuIIIE and DIIIA may inform on the sequence features that drive potency at hNa_V_1.3 over other neuronal subtypes; Similarly, BIIIA and AdIIIA may inform on the sequence features that drive potency at hNa_V_1.2 and hNa_V_1.4, respectively, over other Na_V_ subtypes; Finally, among the seven μ-conotoxins tested in this study, three (BuIIIB, BuIIIE and AdIIIA) were potent blockers of hNa_V_1.7. While not selective for hNa_V_1.7, these μ-conotoxins could potentially offer new insight into the sequence features driving potency at this subtype.

### Supplementary Information

Below is the link to the electronic supplementary material.

Supplementary file1 (PDF 629 KB)

Supplementary file2 (FASTA 17 KB)

## Data Availability

The nucleotide sequences of Bu3.4 (BuIIID), Bu3.8 (BuIIIE), Bu3.5, Bu3.6, Bu3.7, Bu3.9, Bu3.10, Bu3.11, Bu3.12, D3.1, Ad3.1 (including its intronic sequence) and B3.1 have been deposited at DDBJ/EMBL/GenBank [Accessions: OR128446-OR128457]. Sequences of all additional conotoxins identified in the venom gland transcriptome of *C. (T.) bullatus* are provided as Supplementary file 2.
