# Supplementary material for: Identification of sodium channel toxins from marine cone snails of the subgenera Textilia and Afonsoconus
Source: Cell Mol Life Sci. 2023 Sep 9;80(10):287. doi: 10.1007/s00018-023-04935-0 (PMC10492761; doi:10.1007/s00018-023-04935-0)
Supplement: Supplementary file 1 — Supplementary file1 (PDF 629 KB) [file 18_2023_4935_MOESM1_ESM.pdf]

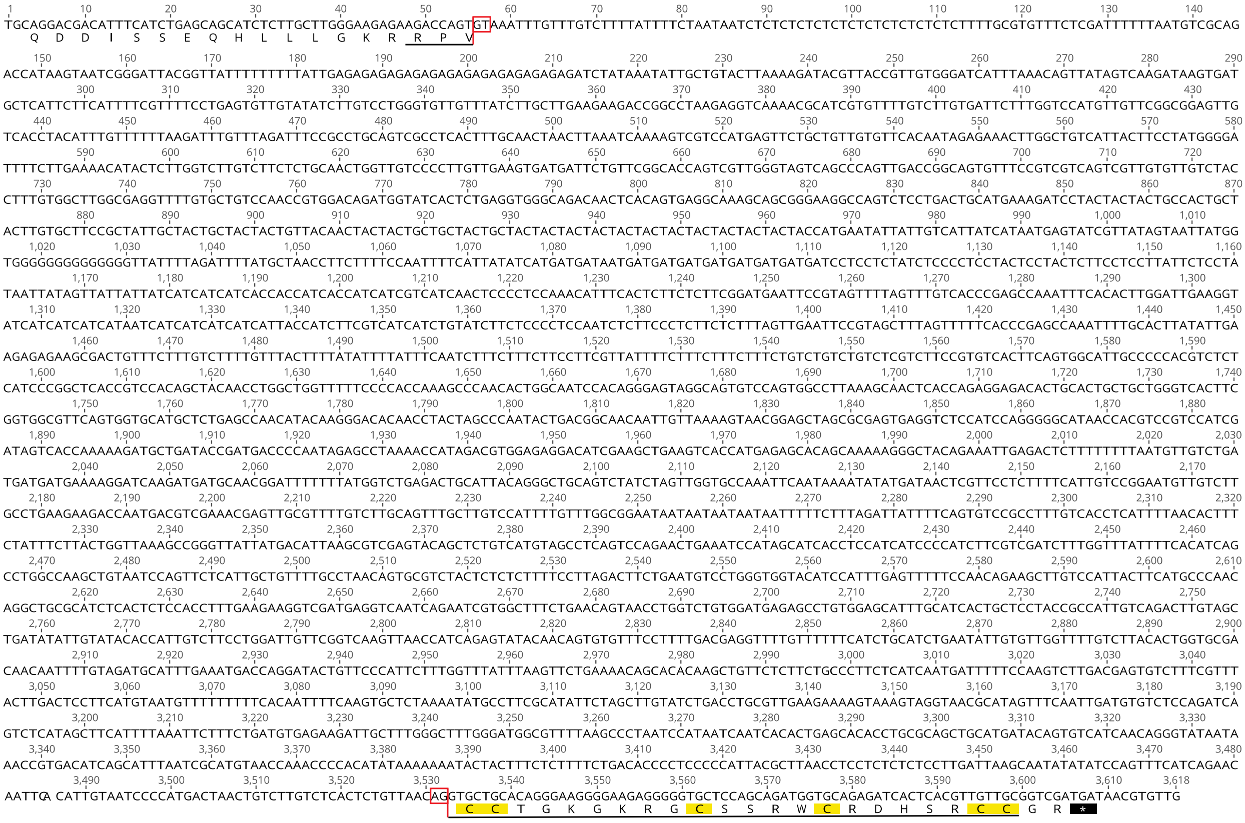

Figure S1. Intron sequence of Ad3.1 (AdIIIA). Translated sequences of exon regions are shown. The predicted mature peptide sequence is underlined. Intron/Exon boundaries are boxed in red; Cysteine residues are coloured yellow.
